# Whole-Genome Sequence of *Corynebacterium pseudotuberculosis* 262 Biovar *equi* Isolated from Cow Milk

**DOI:** 10.1128/genomeA.00176-16

**Published:** 2016-03-24

**Authors:** Carlos Leonardo de A. Araújo, Larissa M. Dias, Adonney A. O. Veras, Jorianne T. C. Alves, Ana Lídia Q. Cavalcante, Christopher G. Dowson, Vasco Azevedo, Rommel T. J. Ramos, Artur Silva, Adriana R. Carneiro

**Affiliations:** a Center of Genomics and System Biology, Laboratory of Genomic and Bioinformatics, Federal University of Pará, Belém, Pará, Brazil; b Institute of Biological Sciences, Federal University of Minas Gerais, Belo Horizonte, Minas Gerais, Brazil; c School of Life Sciences, University of Warwick, Coventry, United Kingdom

## Abstract

We report the complete genome sequence of *Corynebacterium pseudotuberculosis* 262, isolated from a bovine host. *C. pseudotuberculosis* is an etiological agent of diseases with medical and veterinary relevance. The genome contains 2,325,749 bp, 52.8% G+C content, 2,022 coding sequences (CDS), 50 pseudogenes, 48 tRNAs, and 12 rRNAs.

## GENOME ANNOUNCEMENT

*Corynebacterium pseudotuberculosis* is a Gram-positive, facultative intracellular, pleomorphic, nonsporulating, noncapsulated, nonmotile bacterium that is the etiological agent of caseous lymphadenitis (CLA) in small ruminants and pyogranulomatus reactions, ulcerative lymphangitis, and mastitic, necrotic, and ulcerative dermatitis in cattle, all of which are diseases with medical and veterinary relevance. *C. pseudotuberculosis* affects several species, including sheep, goat, horse, cattle, llama, alpaca, buffalo, and human. This organism has various survival mechanisms and uses many strategies to adapt to its environment. After infection, the bacteria become encapsulated within walled-off lesions from which they evade immune system-mediated destruction, giving rise to a state of persistence (1–3). The molecular determinants of *C. pseudotuberculosis* virulence have been described and enable the search for potential targets for the development of new vaccine candidates by “omics” methodologies (4–7).

According to their capability for nitrate reduction, the strains of *C. pseudotuberculosis* are divided into two biovars. The organisms that perform the reduction of nitrate are classified into biovar *equi*, most of which have been isolated from horses and cattle. Bacteria that cannot perform the reduction of nitrate belong to biovar *ovis*, frequently isolated from sheep and goat (8). However, in cattle there are reports of infection by both biovars (9).

Here, we report the genome sequencing of *Corynebacterium pseudotuberculosis* 262, the first strain belonging to biovar *equi* isolated from a bovine host. This strain has been deposited in a collection in Belgium.

*C. pseudotuberculosis* strain 262 was isolated from cow milk, and the genome sequencing was performed with an Ion Torrent PGM platform chip 318, with a fragment library. A total of 388,943,492 bp were produced, with 166× genomic coverage. Subsequently, the tool FastQC 0.11.4 (http://www.bioinformatics.babraham.ac.uk/projects/fastqc/) was used to evaluate the raw data, and FASTX-Toolkit (http://hannonlab.cshl.edu/fastx_toolkit/) was used to remove the reads with quality below Phred 20. The genome assembly was performed by Mira 4.0.2 (http://mira-assembler.sourceforge.net), which produced 29 contigs with an *N*_50_ of 333,604 bp. The manual curation was performed through CLC Genomics Workbench 8 and Artemis 16.0.0 software (10). Automatic genome annotation was performed using Rapid Annotations using Subsystem Technology 2.0 (RAST) (11), and manual curation was performed with Artemis software and the nonredundant protein databases Uniprot (http://www.uniprot.org/) and the National Center for Biotechnology Information (NCBI) (http://www.ncbi.nlm.nih.gov/). tRNAs and rRNAs were predicted using the software tRNAScan-SE 1.21 (12) and RNAmmer 1.2 (13), respectively. The plasticity of pathogenicity islands (PAIs) was assessed with the Pathogenicity Island Prediction Software 1.1 (PIPS) (14), using *C. glutamicum* strain ATCC 21831 (CP007722.1) as the reference genome, which identified 10 pathogenicity islands.

The *C. pseudotuberculosis* strain 262 genome contains 2,325,749 bp, a G+C content of 52.8%, 2,022 coding sequences (CDS), 50 pseudogenes, 48 tRNAs, and 12 rRNAs.

### Nucleotide sequence accession number. 

This whole-genome shotgun project has been deposited at GenBank under accession number CP012022.
